# Characteristic stacked structures and luminescent properties of dinuclear lanthanide complexes with pyrene units

**DOI:** 10.3389/fchem.2023.1154012

**Published:** 2023-04-14

**Authors:** Takuma Nakai, Kaori Shima, Sunao Shoji, Koji Fushimi, Yasuchika Hasegawa, Yuichi Kitagawa

**Affiliations:** ^1^ Graduate School of Chemical Sciences and Engineering, Hokkaido University, Sapporo, Hokkaido, Japan; ^2^ Faculty of Engineering, Sapporo, Hokkaido, Japan; ^3^ Institute for Chemical Reaction Design and Discovery (WPI-ICReDD), Hokkaido University, Sapporo, Hokkaido, Japan

**Keywords:** lanthanide complex, europium, gadolinium, thermometers, luminecence

## Abstract

A novel design strategy of stacked organic fluorophores using dinuclear lanthanide (Ln(III)) complexes is demonstrated for the formation of excimer. The dinuclear Ln(III) complexes are composed of two Ln(III) (Eu(III) or Gd(III)) ions, six hexafluoroacetylacetonate (hfa), and two pyrene-based phosphine oxide ligands. Single-crystal analysis revealed a rigid pyrene-stacked structure via CH-F (pyrene/hfa) intramolecular interactions. The rigid aggregation structures of the two-typed organic ligands around Ln(III) resulted in high thermal stability (decomposition temperature: 340°C). The aggregated ligands exhibited excimer-type green emission from the stacked pyrene-center. The change in the Ln(III) ion promotes effective shifts of excimer emissions (Gd(III):500 nm, Eu(III):490 nm). The organic aggregation system using red-luminescent Eu(III) also provides temperature-sensitive ratiometric emission composed of π-π* and 4f-4f transitions by energy migration between aggregated ligands and Eu(III).

## Introduction

Organic-based luminophores (e.g., organic dyes and metal complexes) are promising materials because of their color-tunable high luminance (Wang et al., 2022a; Wang et al., 2022b). The luminophore design strategy has been studied for the development of displays (Uoyama et al., 2012; Lee et al., 2019; Ha et al., 2021; Hong et al., 2021) and sensors (Rizzo et al., 2004; Merrins et al., 2013; Ni et al., 2020; Fang et al., 2021). Adachi demonstrated efficient organic electroluminescence using thermally assisted delayed fluorescence of organic dyes with a small energy gap between the lowest singlet and triplet excited states (Uoyama et al., 2012). Rizzo developed an organic fluorophore design for effective bio-sensing applications using Förster resonance energy transfer between fluorescent proteins and bio-molecules in living cells (Rizzo et al., 2004; Merrins et al., 2013). Among the reported design strategies, the organic molecular aggregation has become a fascinating approach for luminophore brightness and additional photo-functional properties (Hong et al., 2009).

**FIGURE 1:**
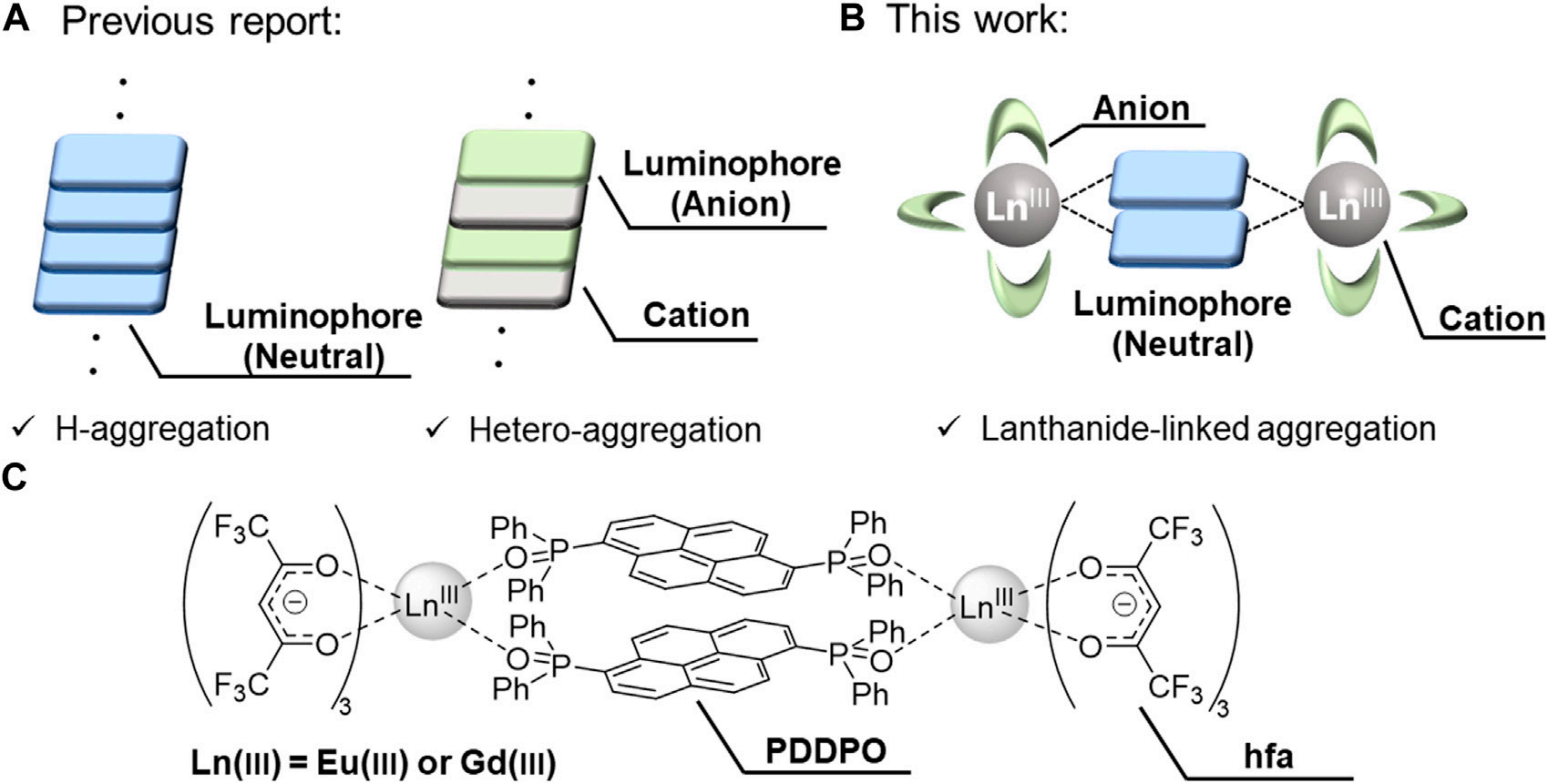
**(A)** Previous strategy: H-aggregation (left) and hetero-aggregation (right). **(B)** This strategy: Lanthanide-linked aggregation. **(C)** Chemical structures of lanthanide complexes.

Tang and co-workers discovered that a pentaphenylsilole fluorophore was nearly non-emissive in dilute solution but exhibited intense emission in the aggregate state, which was named aggregation-induced emission (Luo et al., 2001). This photophysical phenomenon is based on the molecular aggregation structure with ineffective concentration quenching. Huang prepared an H-aggregation composed of 6-diphenyl-2-carbazolyl-1,3,5-triazine, showing ultralong phosphorescence based on an effective stabilization of triplet excited states through strong coupling in the H-type stacked structure (Figure 1A, left) (An et al., 2015). Maeda prepared a regular hetero-aggregation structure composed of chiral anions and a planar triazatriangulenium cation (Figure 1A, right), demonstrating transformable circularly polarized luminescence (CPL) properties (Maeda et al., 2013). Thus, the control of the aggregation structure is a key point for the construction of prominent luminescent materials. Herein, we provide a novel concept for aggregation structures composed of organic fluorophores using lanthanide complexes (Figure 1B).

**FIGURE 2:**
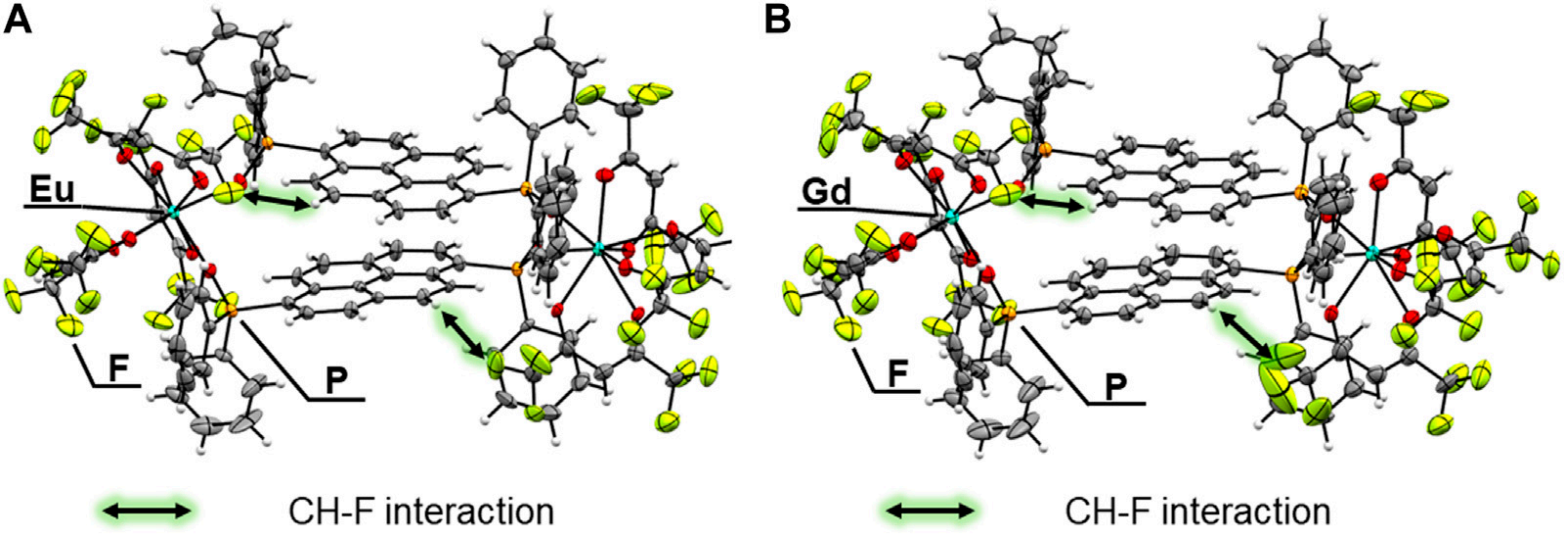
ORTEP drawings (ellipsoids set at 50% probability) of **(A)** [Eu_2_(hfa)_6_(PDDPO)_2_] and **(B)** [Gd_2_(hfa)_6_(PDDPO)_2_].

To demonstrate our strategy, we prepared Ln_2_(hfa)_6_ (hfa: hexafluoroacetylacetonate) and phosphine oxide bridges with a pyrene unit (PDDPO). Non-luminescent Gd(III) and red-luminescent Eu(III) were selected to link the pyrene fluorophores (Figure 1C). The hfa unit and the phosphine oxide ligand containing polyaromatic unit can potentially lead to the formation of a rigid aggregation structure with multiple intramolecular CH–F interactions around the Ln(III) (Figures 2A,B) (Kitagawa et al., 2018; Kitagawa et al., 2020a). The orbital overlap between the heavy metal center and ligand affects the magnitude of the heavy atom effect (Kinoshita et al., 2015; Sasaki et al., 2022). The localized electron population on the pyrene in the PDDPO ligand imparts insignificant internal heavy atom effect from lanthanide ions and reduces the energy loss due to intersystem crossing (Kitagawa et al., 2021a). The characteristic fluorescence properties were derived from a novel organic aggregation system in the lanthanide complexes. The present design for aggregation-type fluorophores should give new insights for the development of luminescent organic materials.

## Experimental

General methods: ^1^H NMR spectra were recorded on a JEOL ECS400 spectrometer. Tetramethylsilane (*δ*_H_ = 0.00 ppm) was used as the internal reference. Electrospray ionization (ESI) mass spectrometry (MS) was performed using a JEOL JMS-T100LP instrument. Elemental analyses were performed on a J-Science Lab JM 10 Micro Corder. Fourier transform infrared (FT-IR) spectra were recorded on a JASCO FT/IR-4600 spectrophotometer. Diffuse-absorption spectra were measured on a JASCO V-670 spectrometer with an integrating-sphere unit (JASCO ISN-723) for Eu(III) and Gd(III) complexes diluted 10,000-folds in KBr. Thermogravimetric analyses (TGA) were performed using a Hitachi High-Tech TG/DTA6300 analyzer under the nitrogen atmosphere at a heating rate of 5 °C min^-1^. Emission spectra (*λ*_ex_ = 325 nm) and excitation spectra (*λ*_em_ = 510 nm) were measured using a Horiba Fluorolog-3 spectrofluorometer. Temperature-dependent emission spectra (*λ*_ex_ = 300 nm) for the Eu(III) complex were measured using a Horiba Fluorolog-3 spectrofluorometer with a cryostat (Thermal Block Company SA-SB245 T) and a temperature controller (Scientific Instruments Model 9700). Emission quantum yields (*λ*_ex_ = 380 nm) were estimated on an FP-6300 spectrofluorometer equipped with an integration sphere (ILF-533). Temperature-dependent emission lifetimes were measured using the third harmonics (*λ*_ex_ = 355 nm) of a Q-switched Nd:YAG laser (Spectra Physics, INDI-50, FWHM = 5 ns, *λ* = 1,064 nm) and a photomultiplier (Hamamatsu Photonics, R5108, response time ≤1.1 ns) with a cryostat (Thermal Block Company SA-SB245 T) and a temperature controller (Scientific Instruments Model 9700). Emission and excitation spectra, emission quantum yields, and emission lifetimes were obtained for Eu(III) and Gd(III) complexes by diluting them 1,000-folds in KBr.

Materials: 1,6-Dibromopyrene (>98.0%), hexafluoroacetylacetone (>95.0%), and chlorodiphenylphosphine (>97.0%) were purchased from Tokyo Chemical Industry Co., Ltd. Tetrahydrofuran, super dehydrated, stabilizer free, 30% hydrogen peroxide, sodium sulfate were purchased from Wako Pure Chemical Industries. Chloroform-*d* (99.8%), *n*-butyllithium in *n*-hexane (1.6 mol/L) were purchased from Kanto Chemical Co., Inc.

Preparation of [pyrene-1,6-diylbis(diphenylphosphine oxide) (PDDPO)]: 1,6-Dibromopyrene (1.0 g, 2.8 mmol) was dissolved in dry tetrahydrofuran (60 ml) and a solution of *n*-butyllithium (3.5 mL, 5.5 mmol) was added to the solution at −76 °C under Ar atmosphere. After 1 h, chloro-diphenylphosphine (1.0 mL, 5.5 mL) was added dropwise to the solution at −76 °C, and then the mixture was gradually brought to room temperature and the solution was stirred for 19 h. The solution was cooled to 0 °C and 30% hydrogen peroxide aqueous solution was added dropwise to the solution. The mixture was stirred for 1.5 h. The reaction mixture was then filtered and poured into the water. The product was extracted using dichloromethane and washed with distilled water. The extract was dried over anhydrous sodium sulfate and evaporated. The compounds were purified by silica gel column chromatography (dichloromethane : acetone = 4 : 1). Yield: 13% (0.22 g, 0.37 mmol). ^1^H NMR (400 MHz, chloroform-*d*, Supplementary Figure S6) δ/ppm = 9.10 (d, *J* = 9.6 Hz, 2H), 8.09 (dd, *J* = 7.2 Hz, 2.4 Hz, 2H), 8.05 (d, *J* = 9.2 Hz, 2H), 7.82–7.63 (m, 10H), 7.61–7.52 (m, 4H), 7.52–7.42 (m, 8H); ESI-MS: m/z calcd. for [C_40_H_29_O_2_P_2_]^+^ = 603.16; found: 603.16.

Preparation of [Eu_2_(hfa)_6_(PDDPO)_2_]: [Eu(hfa)_3_(H_2_O)_2_] (54 mg, 0.066 mmol) and PDDPO (20 mg, 0.033 mmol) were dissolved in dichloromethane (8 mL). The mixture was then stirred for 23 h at room temperature. The solution was recrystallized from dichloromethane and hexane. ESI-MS: m/z calcd. for [C_105_H_61_Eu_2_F_30_O_14_P_4_]^+^ = 2545.10; found: 2545.07. Elemental analysis calcd (%) for C_110_H_62_Eu_2_F_36_O_16_P_4_, C 48.02, H 2.27; found: C 47.58, H 2.04.

Preparation of [Gd_2_(hfa)_6_(PDDPO)_2_]: [Gd(hfa)_3_(H_2_O)_2_] (27 mg, 0.033 mmol) and PDDPO (20 mg, 0.033 mmol) were dissolved in dichloromethane (8 mL). The mixture was then stirred for 2 h at room temperature. The solution was filtered and recrystallized from dichloromethane and hexane. ESI-MS: m/z calcd. for [C_110_H_62_F_36_Gd_2_NaO_16_P_4_]^+^ = 2785.08; found: 2785.07. Elemental analysis calcd (%) for C_110_H_62_F_36_Gd_2_O_16_P_4_, C 47.83, H 2.26; found: C 47.55, H 2.12.

Crystallography: Single crystal X-ray structural analyses for [Eu_2_ (hfa)_6_(PDDPO)_2_] and [Gd_2_(hfa)_6_(PDDPO)_2_] were performed on a Rigaku XtaLAB Synergy-DW diffractometer with graphite monochromatic Mo-Kα radiation (*λ* = 0.71073 Å). Non-hydrogen atoms were refined anisotropically using the SHELX system. All calculations were performed on the crystal structure crystallographic and Olex2 software package. The CIF data were confirmed by the check CIF/PLATON service. CCDC-2238474 (for [Eu_2_(hfa)_6_(PDDPO)_2_]) and CCDC-2235344 (for [Gd_2_ (hfa)_6_(PDDPO)_2_]) contain the supplementary crystallographic data for this paper. These data can be obtained free of charge from The Cambridge Crystallographic Data Centre.

## Results and discussion

Crystal structures: Single crystals of [Eu_2_(hfa)_6_(PDDPO)_2_] and [Gd_2_(hfa)_6_(PDDPO)_2_] were obtained by recrystallization from a mixed solvent of dichloromethane and hexane. The X-ray crystal structure data are presented in Figure 2; Table 1. Space group and crystal system of the Eu(III) complex were determined to be P2_1_/n and monoclinic, respectively. The dinuclear Eu(III) complexes comprised two Eu(hfa)_3_, which were connected by two PDDPO ligands, resulting in the formation of an eight-coordinated dinuclear structure (Figure 2A). The dinculear Eu(III) complex shows a stacked pyrene structure with CH-F interactions (<2.9 Å) between PDDPO and hfa. The crystal structure of the Gd(III) complex is similar to that of the Eu(III) complex (Figure 2B). The distance between exciton centers in the pyrene unit of the Gd(III) complex is the same as that of the Eu(III) complex (*d* = 3.622 Å).

**TABLE 1:** Crystallographic data for [Ln_2_(hfa)_6_(PDDPO)_2_].

	[Eu_2_(hfa)_6_(PDDPO)_2_]	[Gd_2_(hfa)_6_(PDDPO)_2_]
Chemical formula	C_110_H_62_Eu_2_F_36_O_16_P_4_	C_110_H_62_F_36_Gd_2_O_16_P_4_
Formula weight	2751.42	2761.98
Crystal system	monoclinic	monoclinic
Space group	P2_1_/n	P2_1_/n
*a*/Å	12.9730 (4)	13.0128 (3)
*b*/Å	41.8020 (9)	41.8196 (10)
*c*/Å	21.6676 (5)	21.6878 (6)
*α*/deg	90	90
*β*/deg	106.761 (3)	106.721 (3)
*γ*/deg	90	90
Volume/Å	11,251.1 (5)	11,303.3 (5)
*Z*	4	4
Temperature/K	123.15	123.15
*d*_calc_/g cm^-3^	1.624	1.623
*R*_1_	0.0992	0.0678
*wR*_2_	0.2570	0.1698

To clarify the detailed structural differences between Eu(III) and Gd(III) complexes, we performed continuous shape measure (CShM) calculations. The CShM factor (*S*_CShM_) describes the degree of deviation from the ideal structure, and is given by the following equation (Casanova et al., 2005; Das et al., 2016):

$$S_{CShM}=\min\frac{\sum_{k}^{N}{\left|Q_{k}-P_{k}\right|}^{2}}{\sum_{k}^{N}{\left|Q_{k}-P_{0}\right|}^{2}}\times 100$$ (1)

where *N* is the number of vertices, *Q*_0_ is the position vectors of the geometrical center, *Q*_k_ is the vertices of an actual structure, and *P*_k_ is the vertices of an ideal structure. The *S*_CShM_ estimations of the dinuclear complexes revealed a slightly different coordination geometry between the two Eu(III) complex units (Table 2), which correspond to square antiprism (SAP, *S*_SAP_ = 0.589) and trigonal dodecahedron structures (TDH, *S*_TDH_ = 1.066). The Gd(III) dinuclear complex also provided SAP and TDH structures with different *S*_CShM_ values (*S*_SAP_ = 0.634 and *S*_TDH_ = 1.016). CShM analyses indicated the formation of different coordination geometries between Eu(III) and Gd(III) complexes. The angle between the two interplanar pyrene frameworks in the Gd(III) complex (*θ* = 3.08 ^o^) is smaller than that in the Eu(III) complex (*θ* = 3.59 ^o^).

**TABLE 2:** Calculated *S*_CShM_ values for [Ln_2_(hfa)_6_(PDDPO)_2_].

	*S*_SAP_	*S*_TDH_
[Eu_2_(hfa)_6_(PDDPO)_2_]	1.174	1.006
	0.589	2.199
[Gd_2_(hfa)_6_(PDDPO)_2_]	1.185	1.016
	0.634	2.231

**FIGURE 3:**
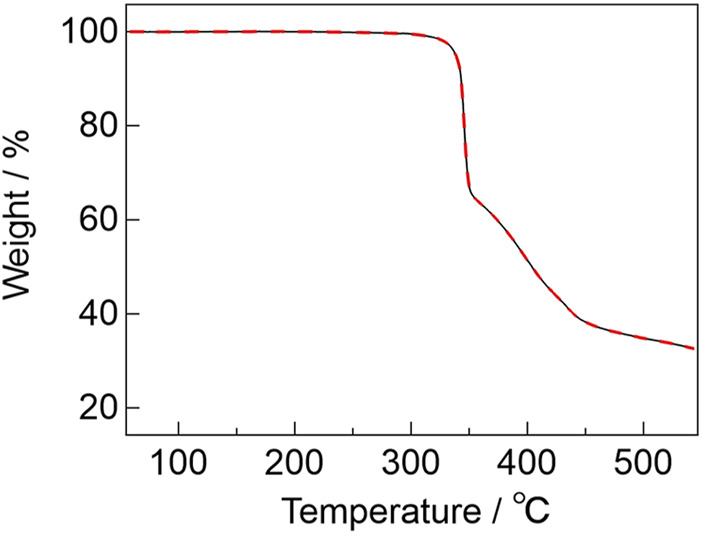
TGA profiles of [Eu_2_(hfa)_6_(PDDPO)_2_] (black line) and [Gd_2_(hfa)_6_(PDDPO)_2_] (red broken line) under a nitrogen atmosphere at a heating rate of 5°C min^-1^.

To evaluate the thermostability of the complexes, thermogravimetric analyses (TGA) were performed for [Eu_2_(hfa)_6_(PDDPO)_2_] and [Gd_2_(hfa)_6_(PDDPO)_2_]. The TGA profiles are shown in Figure 3. The decomposition temperatures of [Eu_2_(hfa)_6_(PDDPO)_2_] and [Gd_2_(hfa)_6_(PDDPO)_2_] were determined to be 340 °C. Their high thermostabilities originate from rigid aggregation based on intramolecular CH-F interactions.

**FIGURE 4:**
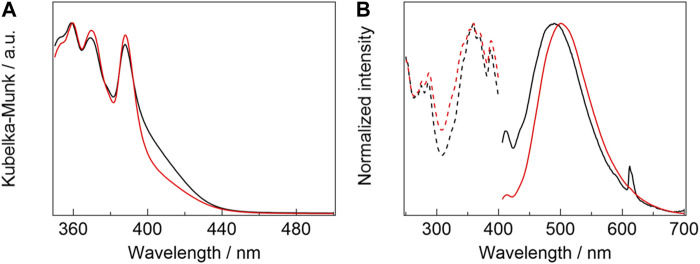
**(A)** Diffuse-absorption spectra of [Eu_2_(hfa)_6_(PDDPO)_2_] (black line) and [Gd_2_(hfa)_6_(PDDPO)_2_] (red line). **(B)** Emission (solid line, *λ*_ex_= 325 nm) and excitation spectra (broken line, *λ*_em_= 510 nm) of [Eu_2_(hfa)_6_(PDDPO)_2_] (black line) and [Gd_2_(hfa)_6_(PDDPO)_2_] (red line).

Photophysical properties: Diffuse-absorption spectra of [Eu_2_(hfa)_6_(PDDPO)_2_] and [Gd_2_(hfa)_6_(PDDPO)_2_] are shown in Figure 4A. Absorption bands are observed at 388, 369, and 359 nm for [Gd_2_(hfa)_6_(PDDPO)_2_]. Their absorption bands are assigned to (0, 0), (0, 1), and (0, 2) π-π* transitions of the PDDPO ligands, respectively [Eu_2_(hfa)_6_(PDDPO)_2_] exhibits an additional shoulder band at around 420 nm, which is assigned to a ligand-to-metal charge transfer (LMCT) transition. The LMCT band positions can be understood by comparing the diffuse-absorption spectra of the Eu(III) and Gd(III) complexes (Kitagawa et al., 2020b). Eu(III) has a low reduction potential (Eu(III)/Eu(II) = −0.35 V vs NHE) (MacDonald et al., 2013), resulting in low-energy LMCT states for Eu(III) complexes. On the other hand, Gd(III) complexes show high-energy LMCT states due to the high reduction potential of Gd(III) ions (Gd(III)/Gd(II) = −3.9 V vs NHE) (MacDonald et al., 2013), resulting in high-energy LMCT states for Gd(III) complexes. The LMCT band positions can be understood by comparing the diffuse-absorption spectra of the Eu(III) and Gd(III) complexes.

Emission spectra of [Eu_2_(hfa)_6_(PDDPO)_2_] and [Gd_2_(hfa)_6_(PDDPO)_2_] are shown in Figure 4B (solid line). The spectra are normalized at their intensity maxima. A weak emission band is observed at 413 nm for [Gd_2_(hfa)_6_(PDDPO)_2_], which might be attributed to π*→π transition of the pyrene unit in the dinculear complex (Wang et al., 2017). A strong and broad emission band is also observed at 500 nm (full-width half maximum (FWHM): 98 nm, *τ* = 6.9 ns) for [Gd_2_(hfa)_6_(PDDPO)_2_]. The excitation spectra for the broad emission band (*λ*_em_ = 510 nm) correspond to the transition of the monomeric pyrene unit (Figure 4B, broken line). To clarify the broad emission band, emission spectrum of free PDDPO ligand was measured (Supplementary Figure S8). Emission bands at 427, 405, and 385 nm, which are originated from π*→π transition of PDDPO ligand, are observed. These results indicate that the broad emission band is attributed to the formation of pyrene excimer in the Gd(III) complex [Eu_2_ (hfa)_6_(PDDPO)_2_] also provides sharp and broad emission bands at 413 and 490 nm (FWHM: 107 nm, *τ* = 12 ns), which are assigned to the emission bands from monomeric and excimer pyrene units, respectively. In addition, a sharp emission band is observed at 612 nm for [Eu_2_(hfa)_6_(PDDPO)_2_], which is assigned to ^5^D_0_→^7^F_2_ transition of Eu(III) (Kitagawa et al., 2018). The phosphorescence spectrum of [Gd_2_(hfa)_6_(PDDPO)_2_] was measured to clarify the T_1_ level using the time-resolved emission measurement technique (Supplementary Figure S9). From the measurements, the T_1_ level of pyrene excimer state in the lanthanide complex was estimated to be 14,600 cm^-1^. The energy diagram for the Eu(III) complex is shown in Supplementary Figure S11. The estimated T_1_ level indicates ineffective energy transfer from PDDPO to Eu(III) (^5^D_0_: 17,200 cm^-1^) (Bao et al., 2018). According to a previous report, hfa (S_1_ = 24,000 cm^-1^, T_1_ = 21,700 cm^-1^) (Yamamoto et al., 2018) allows highly efficient energy transfer to Eu(III) ion (Moudam et al., 2009; Shoji et al., 2022). Thus, the Eu(III) emission (*λ*_ex_ = 325 nm) is considered to originate from the energy transfer from the hfa ligands. The emission quantum yields (*Φ*) of [Gd_2_(hfa)_6_(PDDPO)_2_] and [Eu_2_(hfa)_6_(PDDPO)_2_], mainly from the excited PDDPO ligands (*λ*_ex_ = 380 nm in Ar), were estimated to be 27.6% ± 0.47% and 3.99% ± 0.31%, respectively. The results suggest that the excimer emission in the Eu(III) complex is quenched by energy accepting states of Eu(III) (^5^D_0_ and/or ^5^D_1_). The excimer emission band of [Eu_2_(hfa)_6_(PDDPO)_2_] was blue-shifted by 10 nm blue-shifted compared to that of [Gd_2_(hfa)_6_(PDDPO)_2_]. A previous study demonstrates an example of large emission color shift in a pyrene excimer upon changing the substituents of pyrene (Ge et al., 2020). Considering the crystal structure, the blue-shifted excimer emission originates from the different orientations of the pyrene units between [Gd_2_(hfa)_6_(PDDPO)_2_] and [Eu_2_(hfa)_6_(PDDPO)_2_]. The lanthanide-linked excimer system is a novel strategy for effective control of the emission wavelength by changing the lanthanide ion.

Finally, we evaluated temperature-sensitive emission properties using a Ln(III)-linked aggregation system to demonstrate thermometer applications. Luminescent molecular thermometers have attracted attention in the fields of aeronautical science, microelectronics, and environment engineering (Wang et al., 2013; Chan et al., 2021). Among them, ratiometric luminescent thermometers are not sensitive to variations in luminophore concentration, surrounding environment, and sample viscosity. The present Eu(III)-linked aggregation system is a promising candidate for ratiometric luminescent thermometers.

**FIGURE 5:**
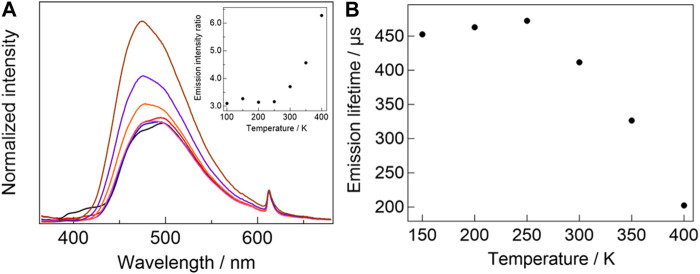
**(A)** Temperature-dependent emission spectra of [Eu_2_(hfa)_6_(PDDPO)_2_] (black line: 100 K, red line: 150 K, blue line: 200 K, pink line: 250 K, orange line: 300 K, purple line: 350 K, brown line: 400 K, *λ*_ex_= 300 nm). Inset: temperature-dependent emission intensity ratio of pyrene excimer to Eu(III). **(B)** Temperature-dependent emission lifetime of [Eu_2_(hfa)_6_(PDDPO)_2_].

The temperature-dependent emission spectra of [Eu_2_ (hfa)_6_(PDDPO)_2_] are shown in Figure 5A. The spectra are normalized at 612 nm with respect to the 4f-4f emission bands to clarify the ratiometric emission properties. The pyrene excimer emission band is slightly blue-shifted from 498 nm (100 K) to 474 nm (400 K) upon increasing the temperature, which originates from the stacked structure change of the two pyrene units depending on the temperature (Shen et al., 2019). The Eu(III) complex shows temperature-sensitive ratiometric emission composed of excimer π-π* and Eu(III) 4f-4f emission bands. The emission intensity ratio of the excimer to Eu(III) increases with increased temperature in the range of 250–400 K (Figure 5A, inset). To evaluate the performance as the temperature sensor of [Eu_2_ (hfa)_6_(PDDPO)_2_], the relative sensitivity (*S*_r_) was evaluated by following equation (Zhang et al., 2019):

$$S_{r}=\left|\frac{1}{FIR}\frac{\partial \left(FIR\right)}{\partial T}\right|\times 100\%$$ (2)

where FIR is the fluorescence intensity ratio. The emission intensity ratio of the excimer (*λ* = 500 nm) to Eu(III) (*λ* = 612 nm) was selected for the analysis. The value of maximum relative sensitivity (*S*_m_) was estimated to be 0.75% K^−1^ (350 K).

To investigate the temperature-sensitive emission mechanism, temperature-dependent Eu(III) emission lifetimes of [Eu_2_ (hfa)_6_(PDDPO)_2_] (Figure 5B) were measured. The emission lifetime of the Eu(III) complex decreases with increasing temperature in the range of 250–400 K, which is consistent with the temperature range for ratiometric emission. These results indicate the occurrence of back energy transfer (BEnT) from Eu(III) ^5^D_0_ state to ligand-aggregation system (Kitagawa et al., 2020c; Kitagawa et al., 2021b). Here, the BEnT rate constants (*k*_BEnT_) were estimated using kinetic analysis. The Arrhenius-type equation is expressed as follows (Miyata et al., 2013):

$$\ln\left(\frac{1}{\tau_{obs}}-\frac{1}{\tau_{const}}\right)=\ln\left(k_{BEnT}\right)=\ln A-\frac{E_{a}}{R}\times T^{-1}$$ (3)

where *A* is the frequency factor and *E*_a_ is the activation energy. *A* and *E*_a_ were estimated to be 2.9 × 10^6^ s^-1^ and 1,950 cm^-1^, respectively. TD-DFT calculation was performed for a stacked pyrene structure (Supporting Material) to determine the energy quenching state for ^5^D_0_ emission in [Eu_2_(hfa)_6_(PDDPO)_2_]. The estimated T_1_, T_2_, and T_3_ energy levels were 15,522, 16,765, and 24,387 cm^−1^, respectively (Supplementary Table S1). These values do not match the energy level of the quenching state assumed by the activation energy (1,950 cm^−1^) and emitting level of Eu(III) (17,200 cm^−1^) (Yamamoto et al., 2018). The large energy gap between hfa T_1_ and ^5^D_0_ states (4,900 cm^−1^) results in ineffective BEnT from ^5^D_0_ to the hfa T_1_ state. The stacked pyrene structure provides a high HOMO level (−5.05 eV, Supplementary Figure S2), resulting in the formation of an LMCT state with low energy level (Kitagawa et al., 2020b). We consider that the BEnT from ^5^D_0_ to the LMCT excited state in the Eu(III) complex is a key factor for the ratiometric luminescent property. To the best of our knowledge, this is the first example of ratiometric luminescent thermometer composed of organic excimer and lanthanide emission.

## Conclusion

We prepared novel dinuclear Eu(III) and Gd(III) complexes with stacked pyrene structures. The change in the lanthanide(III) linker resulted in the control of the stacked pyrene structures and their excimer emission wavelength (Eu(III): 490 nm, Gd(III): 500 nm). The stacked pyrene structure with luminescent Eu(III) linker also provided a ratiometric luminescent thermometer property composed of excimer π-π* emission and Eu(III) 4f-4f emission (*S*_m_ = 0.75% K^−1^). This novel design concept of aggregation structure control opens up a frontier field of material science, photophysics, and coordination chemistry.

## Data Availability

The datasets presented in this study can be found in online repositories. The names of the repository/repositories and accession number(s) can be found below: https://www.ccdc.cam.ac.uk/structures/ - 2238474 and 2235344.
