# Supplementary material for: Characteristic stacked structures and luminescent properties of dinuclear lanthanide complexes with pyrene units
Source: Front Chem. 2023 Apr 14;11:1154012. doi: 10.3389/fchem.2023.1154012 (PMC10140548; doi:10.3389/fchem.2023.1154012)
Supplement: Supplementary file 1 [file DataSheet1.pdf]

*Supplementary Material*

# Characteristic stacked structures and luminescent properties of dinuclear lanthanide complexes with pyrene units

**Takuma Nakai[1], Kaori Shima[1], Sunao Shoji[2,3], Koji Fushimi[2], Yasuchika Hasegawa*[2,3], and Yuichi Kitagawa*[2,3]**

[1]Graduate School of Chemical Sciences and Engineering, Hokkaido University, Kita 13, Nishi 8, Kita-ku, Sapporo, Hokkaido, 060-8628, Japan.

[2]Faculty of Engineering, Kita 13, Nishi 8, Kita-ku, Sapporo, Hokkaido, 060-8628, Japan.

[3]Institute for Chemical Reaction Design and Discovery (WPI-ICReDD), Hokkaido University, Kita 21, Nishi 10, Kita-ku, Sapporo, Hokkaido, 001-0021, Japan.

*** Correspondence:**

Yuichi Kitagawa: y-kitagawa@eng.hokudai.ac.jp

Yasuchika Hasegawa: hasegaway@eng.hokudai.ac.jp

**TD-DFT calculation**

To investigate the excited state of $[Eu_2(hfa)_6(PDDPO)_2]$, time-dependent density functional theory (TD-DFT) calculation using B3LYP functional was performed. To simplify the models, stacked PDDPO ligands in $[Eu_2(hfa)_6(PDDPO)_2]$ were extracted (Fig. S1). The 6-31G(D) basis set was adopted.

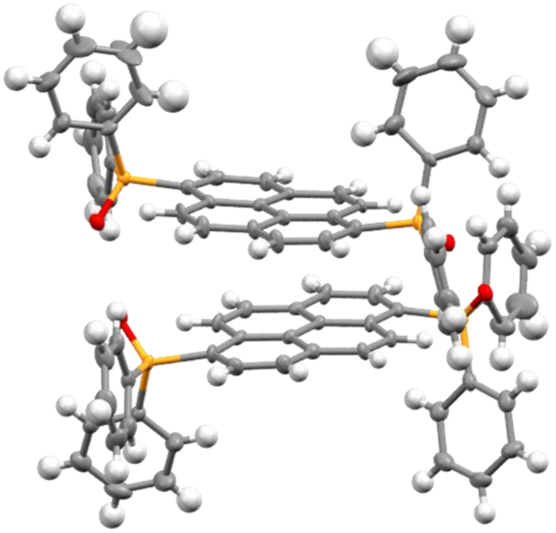

**FIGURE S1.** Stacked PDDPO ligands in $[Eu_2(hfa)_6(PDDPO)_2]$

**Table S1.** Wavelength, Energy levels, and main configurations of $T_1$, $T_2$, $S_1$, and $T_3$ for stacked pyrene structures.

| Excited state | Wavelength / nm | Energy / $cm^{-1}$ | Main configuration |
|---|---|---|---|
| $T_1$ | 644.23 | 15,522 | HOMO → LUMO |
| $T_2$ | 596.45 | 16,765 | HOMO → LUMO+1 |
| $S_1$ | 428.70 | 23,326 | HOMO → LUMO |
| $T_3$ | 410.05 | 24,387 | HOMO → LUMO+1 |

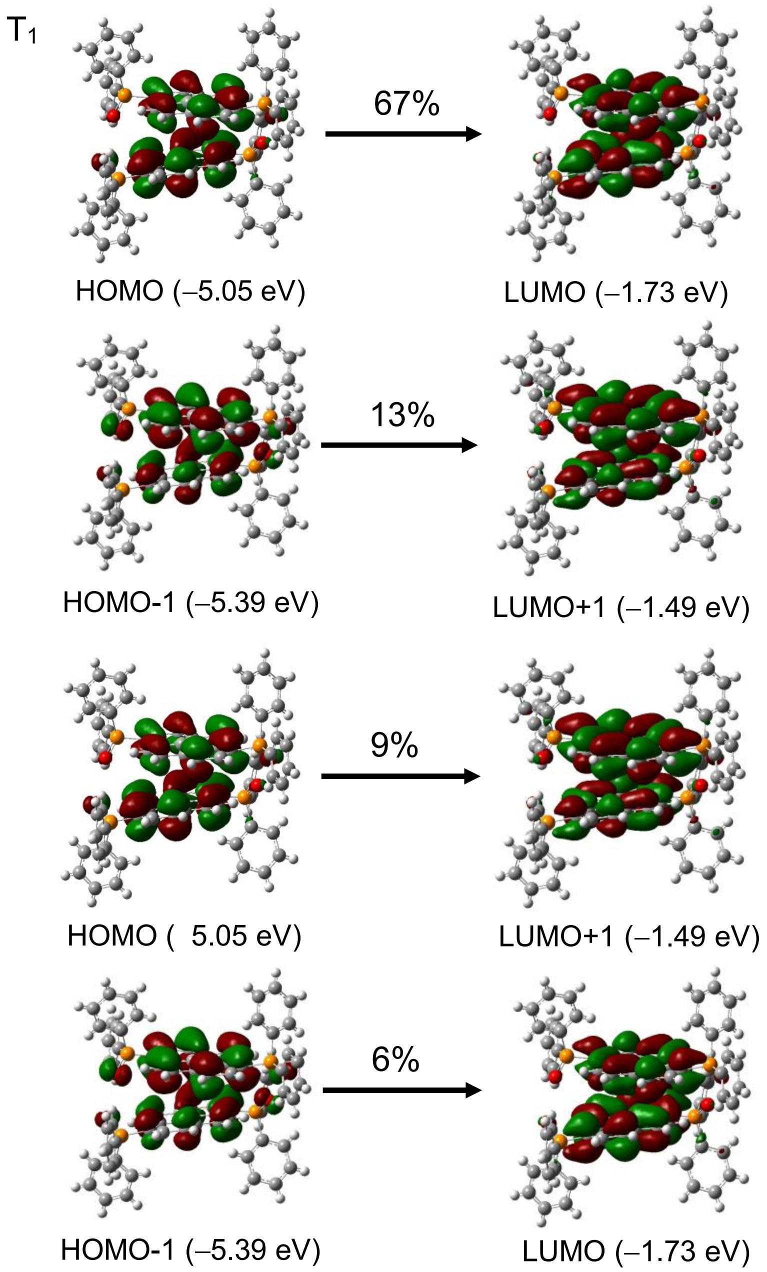


**FIGURE S2.** Main electronic configurations of $S_0$-$T_1$ transitions for stacked pyrene structures.

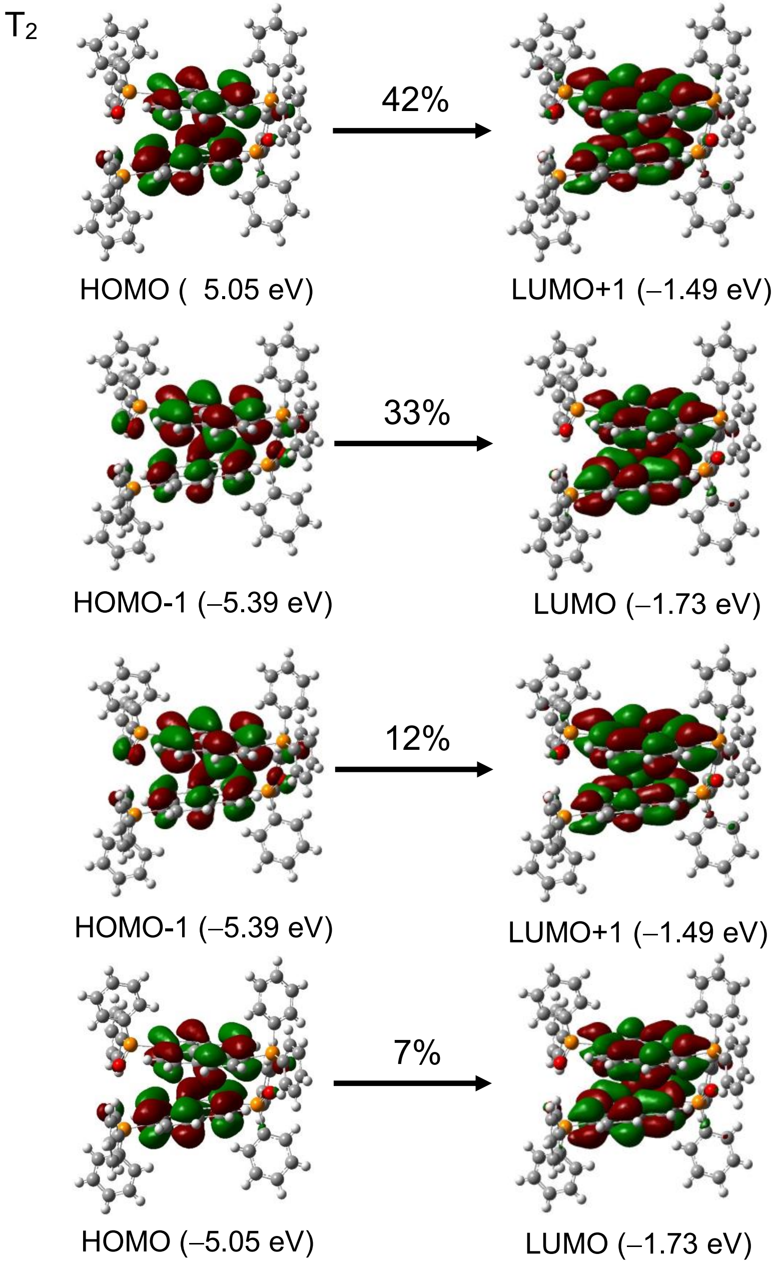


**FIGURE S3.** Main electronic configurations of $S_0$-$T_2$ transitions for stacked pyrene structures.

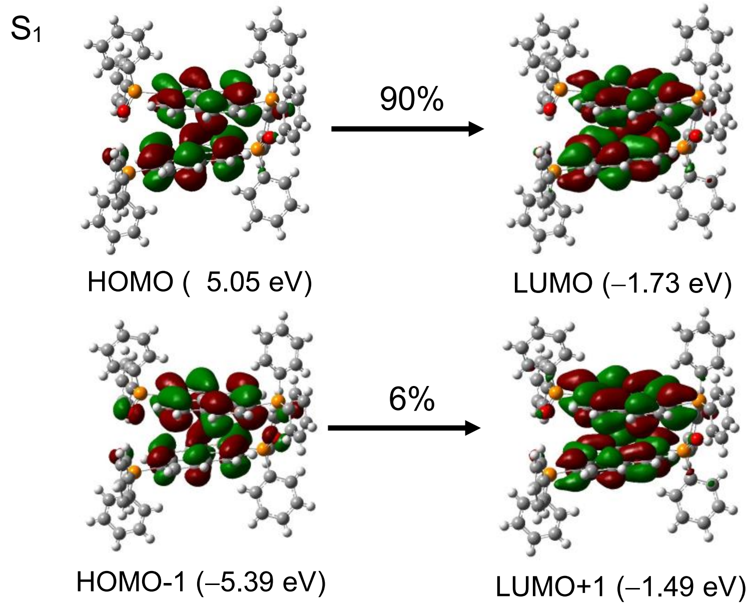


**FIGURE S4.** Main electronic configurations of $S_0$-$S_1$ transitions for stacked pyrene structures.

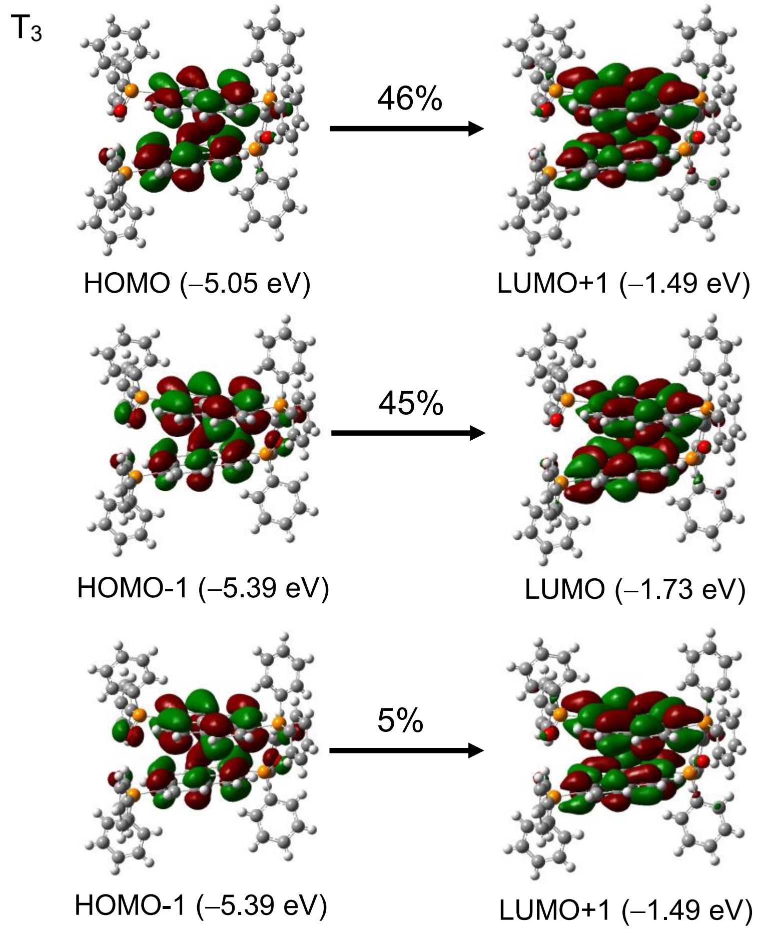


**FIGURE S5.** Main electronic configurations of $S_0$-$T_3$ transitions for stacked pyrene structures.

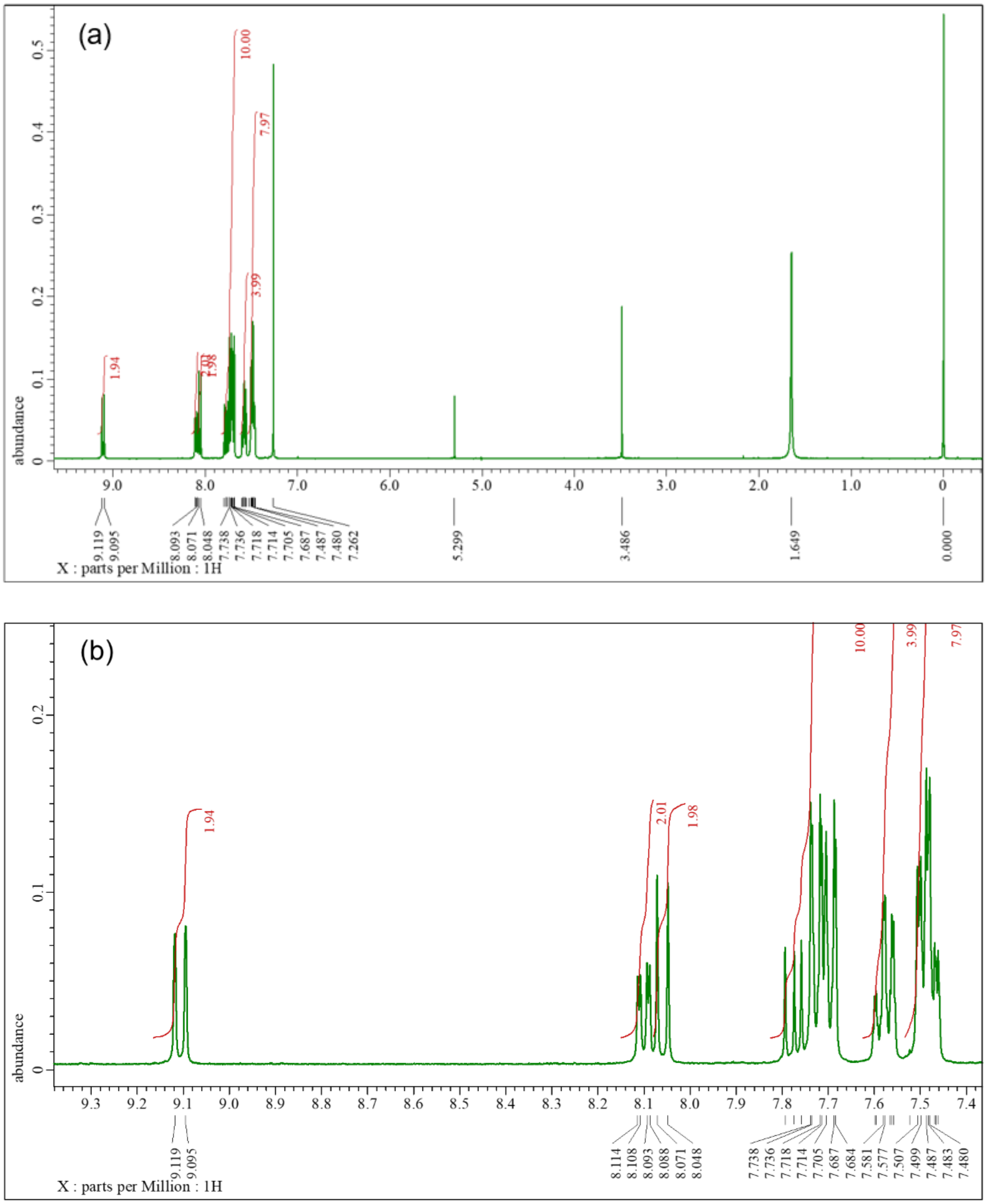


**FIGURE S6.** $^{1}$H NMR spectra of PDDPO in chloroform-*d*

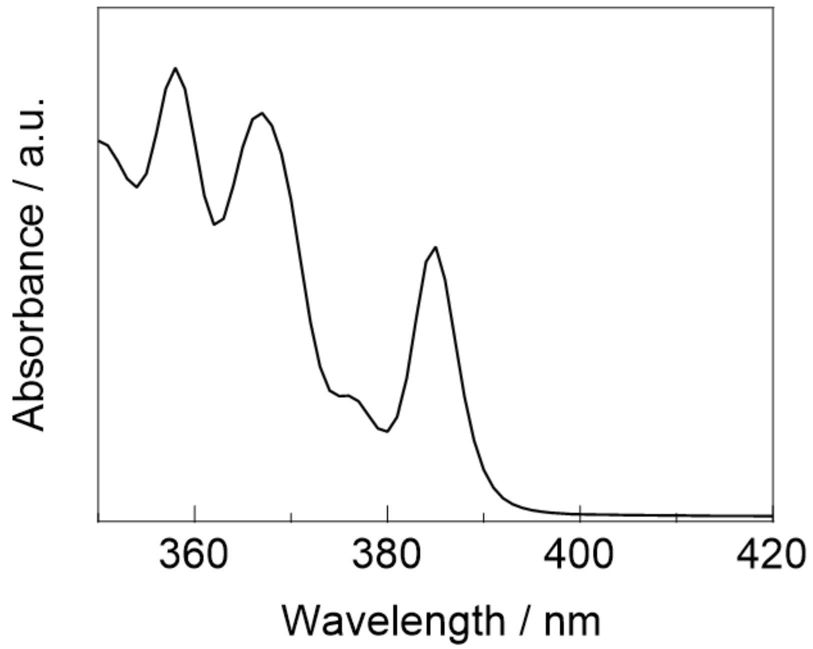


**FIGURE S7.** Absorption spectrum of PDDPO in dichloromethane (1.0×$10^{-4}$ M)

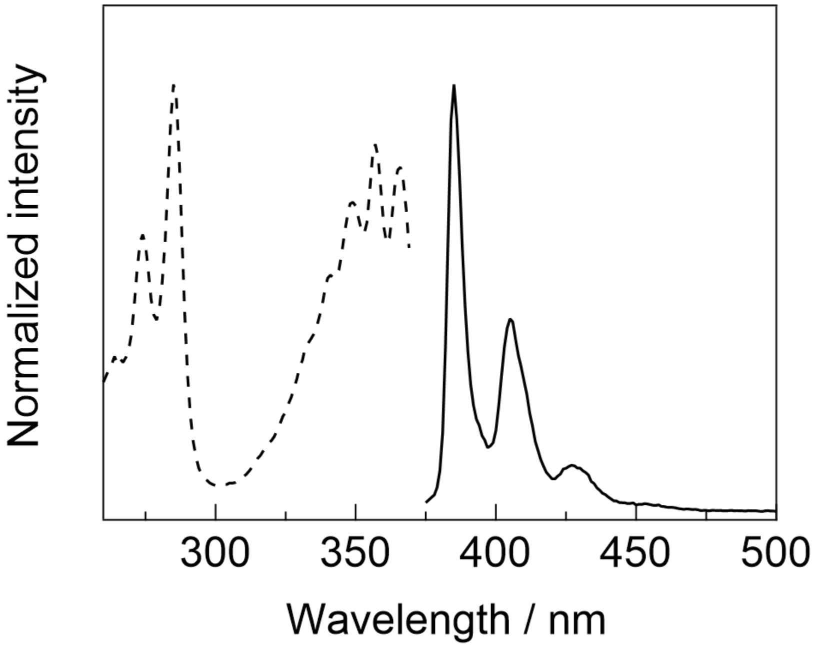


**FIGURE S8.** Emission (solid line, $\lambda_{ex}$= 325 nm) and excitation spectra (broken line, $\lambda_{em}$= 450 nm) of PDDPO (black line) in dichloromethane (1.0×$10^{-5}$ M).

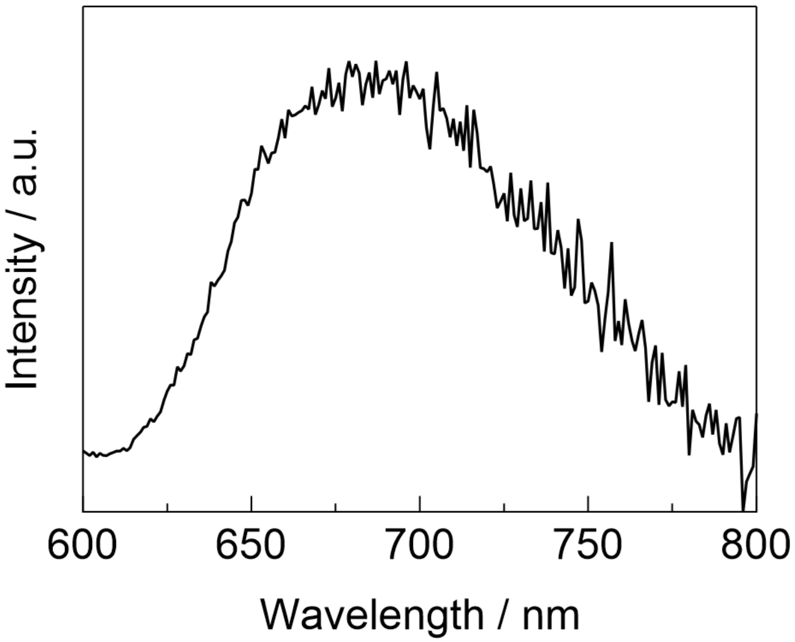


**FIGURE S9.** Phosphorescence spectrum of [$Gd_2(hfa)_6(PDDPO)_2$] ($\lambda_{ex}$ = 380 nm; delay: 20 ms, 100 K).

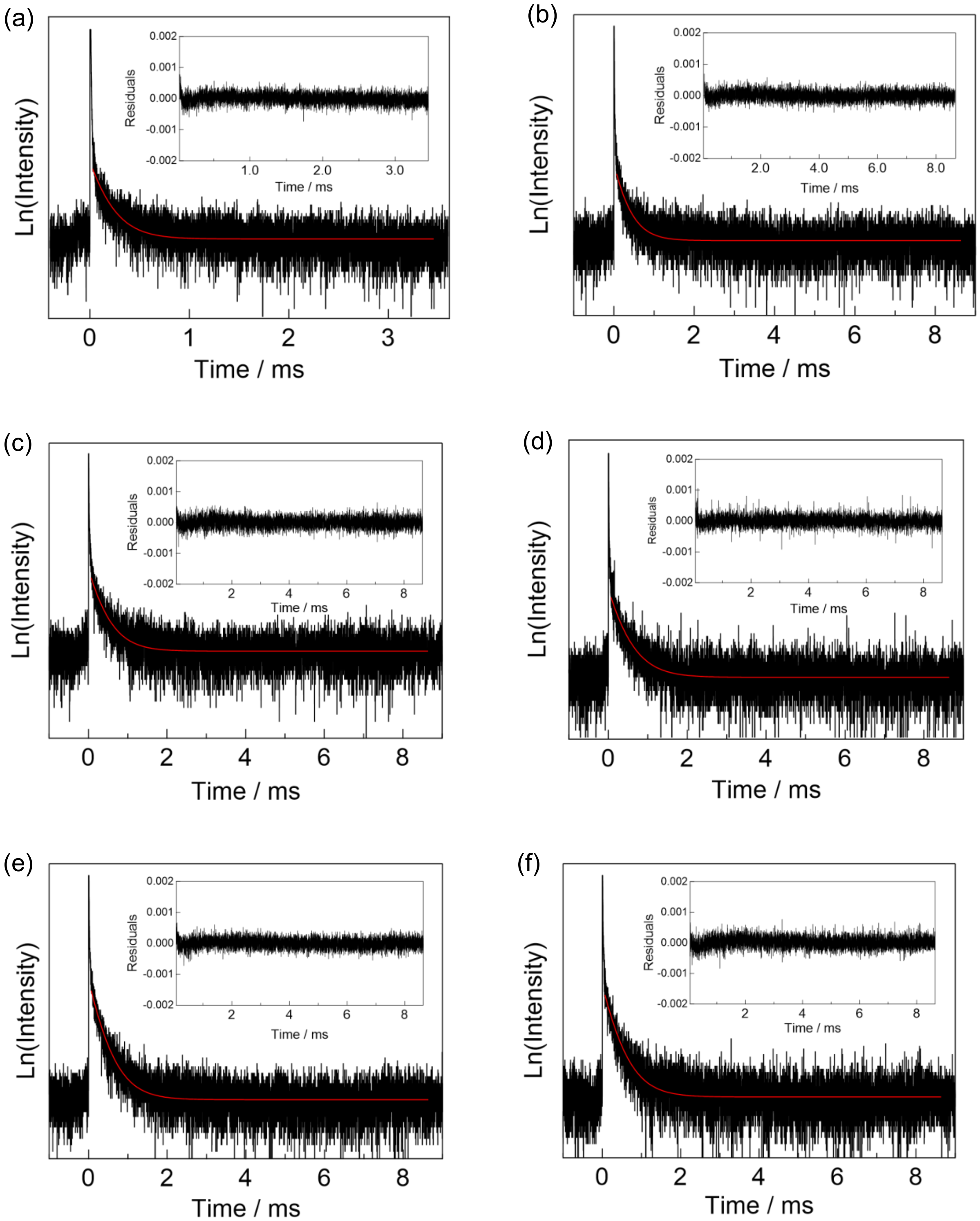


**FIGURE S10.** Emission decay curves of [$Eu_2(hfa)_6(PDDPO)_2$] (a: 400 K, b: 350 K, c: 300 K, d: 250 K, e: 200 K, f: 150 K). Inset: Residuals between emission decay curve (black) and fitting curve (red line).

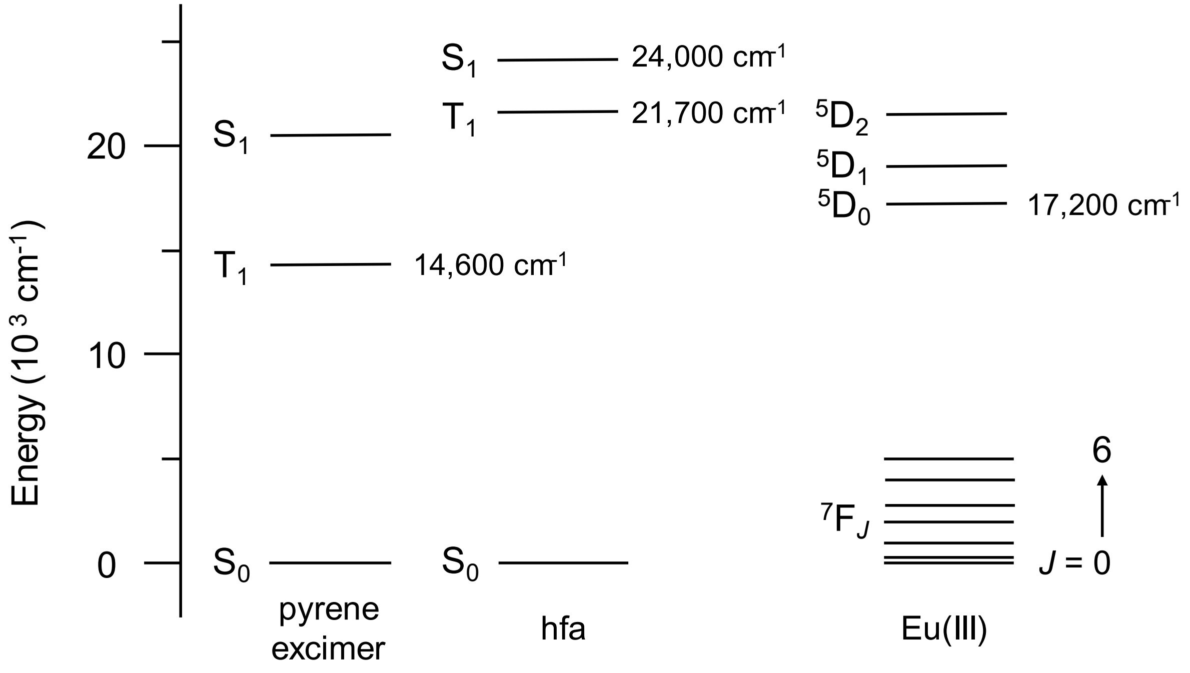


**FIGURE S11.** Energy diagram for $[Eu_2(hfa)_6(PDDPO)_2]$.

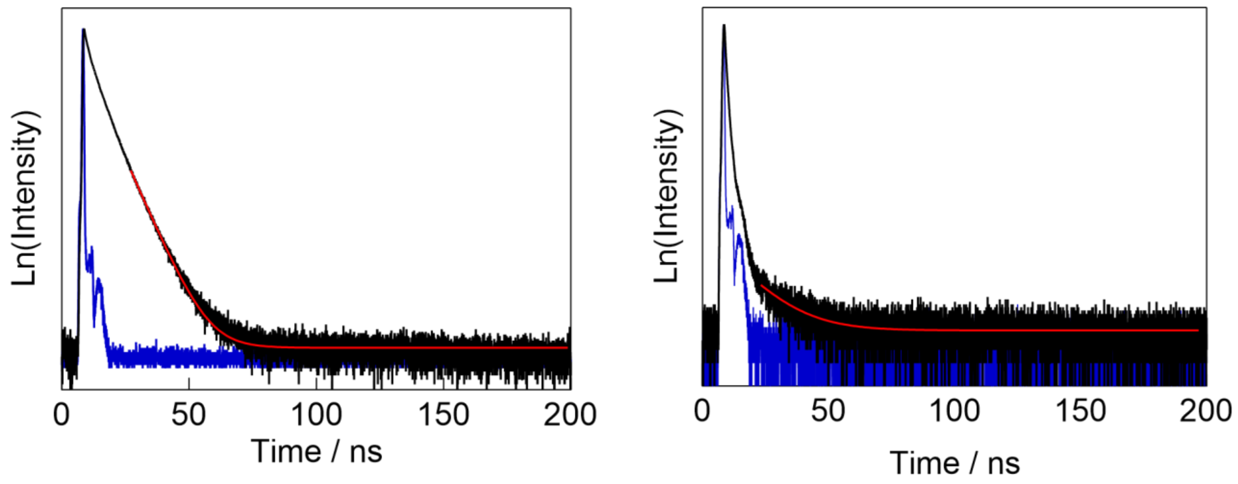


**FIGURE S12.** Emission decay curves of $[Gd_2(hfa)_6(PDDPO)_2]$ (left, $\lambda_{em}$ = 500 nm) and $[Eu_2(hfa)_6(PDDPO)_2]$ (right, $\lambda_{em}$ = 490 nm) in solid states using FLS1000 with EPL-UV picosecond pulsed diode laser ($\lambda_{ex}$ = 375 nm). Blue lines and red lines in the figure show IRF and fitting curve, respectively.
